# Justification of Indication for Cranial CT Imaging after Mild Traumatic Brain Injury According to the Current National Guidelines

**DOI:** 10.3390/diagnostics13111826

**Published:** 2023-05-23

**Authors:** Andreas Sakkas, Christel Weiß, Frank Wilde, Marcel Ebeling, Mario Scheurer, Oliver Christian Thiele, Robert Andreas Mischkowski, Sebastian Pietzka

**Affiliations:** 1 Department of Cranio-Maxillo-Facial-Surgery, University Hospital Ulm, 89081 Ulm, Germany; 2 Department of Cranio-Maxillo-Facial-Surgery, German Armed Forces Hospital Ulm, 89081 Ulm, Germany; 3 Medical Statistics and Biomathematics, University Medical Centre Mannheim, Heidelberg University, 68167 Mannheim, Germany; 4 Department of Oral and Plastic Maxillofacial Surgery, Ludwigshafen Hospital, 67063 Ludwigshafen, Germany; octhiele@yahoo.de (O.C.T.);

**Keywords:** intracranial hemorrhage, computer tomography, mild traumatic brain injury, decision rules

## Abstract

The primary aim was to evaluate the compliance of cranial CT indication with the national guideline-based decision rules in patients after mTBI. The secondary aim was to determine the incidence of CT pathologies among justified and unjustified CT scans and to investigate the diagnostic value of these decision rules. This is a retrospective, single-center study on 1837 patients (mean age = 70.7 years) referred to a clinic of oral and maxillofacial surgery following mTBI over a five-year period. The current national clinical decision rules and recommendations for mTBI were retrospectively applied to calculate the incidence of unjustified CT imaging. The intracranial pathologies among the justified and unjustified CT scans were presented using descriptive statistical analysis. The performance of the decision rules was ascertained by calculating the sensitivity, specificity, and predictive values. A total of 123 intracerebral lesions were radiologically detected in 102 (5.5%) of the study patients. Most (62.1%) of the CT scans strictly complied with the guideline recommendations, and 37.8% were not justified and likely avoidable. A significantly higher incidence of intracranial pathology was observed in patients with justified CT scans compared with patients with unjustified CT scans (7.9% vs. 2.5%, *p* < 0.0001). Patients with loss of consciousness, amnesia, seizures, cephalgia, somnolence, dizziness, nausea, and clinical signs of cranial fractures presented pathologic CT findings more frequently (*p* < 0.05). The decision rules identified CT pathologies with 92.28% sensitivity and 39.08% specificity. To conclude, compliance with the national decision rules for mTBI was low, and more than a third of the CT scans performed were identified as “likely avoidable”. A higher incidence of pathologic CT findings was detected in patients with justified cranial CT imaging. The investigated decision rules showed a high sensitivity but low specificity for predicting CT pathologies.

## 1. Introduction

Traumatic brain injury (TBI) is an intense neurologic disorder with a significant financial impact on health systems [1]. Mild TBI (mTBI) as the most common type is defined by an initial Glasgow Coma Scale (GCS) score of ≥13, posttraumatic loss of consciousness of <30 min, posttraumatic amnesia of <24 h and mental alteration, or other focal neurological deficits [2,3,4].

Cranial computer tomography (CT) has been proven to be an effective modality for diagnosis and intervention following mTBI [5,6]. However, knowing which patients with this injury pattern could benefit from CT imaging still poses a challenge in decision-making for physicians of various disciplines. Fear of the consequences of delayed or inadequate therapeutic management after mTBI, especially in patients with risk factors, has led to routine CT overuse in emergency departments, which is not always justified by the recommended clinical criteria [6,7,8,9,10,11,12,13]. Consequently, extensive CT use leads to unnecessary exposure to ionizing radiation, time loss, and ineffective cost expansion. Thus, better imaging stewardship is required to decrease the hospitalization length and improve the level of emergency care [14,15].

Multiple validated clinical decision rules for detecting intracranial injuries have been developed in the past to standardize and increase the efficiency of CT indication after mTBI [6,8,11,16,17]. The 2001-established Canadian CT Head Rule (CCHR) was the most common rule, and externally validated and highly sensitive, but with variation in its specificity [18,19,20,21]. Other decision criteria, such as New Orleans, NICE, Scandinavian, and NEXUS-II, have demonstrated their validity and generalizability based on different clinical and demographic indicators for CT imaging [15,21]. A discussed weakness of these decision rules is that are not validated for patients under antithrombotic therapy [9,11,21,22,23,24,25]. Considering the widespread use of new antithrombotic medications in recent years, the correct management of this patient cohort after mTBI has gained increased interest from researchers.

According to the current German guidelines for mTBI patients, a cranial CT is indicated as the “gold standard” in patients with coma or in patients who have experienced a loss of consciousness, definite amnesia, multiple vomiting, seizures, pathologic neurological signs, clinical signs of cranial fracture, suspicion of impression fracture or penetrating injuries, suspicion of liquorrhea, coagulopathy, or the use of antithrombotic medication [2]. An optional indication exists in case of severe cephalgia, alcohol or drug intoxication, unclear information about the trauma mechanism, and high-energy trauma [2]. Considering the risk−benefit assessment for the patient and the institution, the investigation of unjustified cranial CT scans in mTBI patients is lacking in Germany. Determining the incidence of likely avoidable CT imaging could be essential for reducing unjustified CT imaging usage. To the best of our knowledge, no previous study has investigated the justification of cranial CT indication after mTBI in a clinic of oral and maxillofacial surgery in Germany that applies the national mTBI guidelines as the treatment standard.

The primary aim of this study was to evaluate the compliance of cranial CT indication with the national guideline-based decision rules in patients referred to an oral and maxillofacial surgery clinic after mTBI, and thus determine the incidence of unjustified CT scans by retrospectively reapplying the guidelines’ recommendations. The secondary aim was to determine the incidence of CT pathologies among justified and unjustified CT scans. The association of clinical decision rule implementation with diagnostic effectiveness was examined by calculating the sensitivity, specificity, and positive and negative predictive values to increase imaging appropriateness. We hypothesized a low compliance resulting in unnecessary CT imaging.

## 2. Materials and Methods

### 2.1. Patient Collection

For this observational retrospective single-center study, medical records of all patients who were referred to our emergency department for oral and plastic maxillofacial surgery with mTBI trauma between January 2016 and December 2020 were reviewed. Records were retrieved from the hospital electronic database. Ethical approval for this research was obtained from the ethics committee of the chamber of physicians in Rhineland-Palatine, Mainz, Germany, in the context of a large retrospective trauma data evaluation with different aims and variable scientific issues (approval number: 2018-13524) [3,4].

Patients who met the following inclusion criteria were enrolled: (1) full age, (2) head trauma, (3) GCS score 13–15 at admission, (4) loss of consciousness for less than 30 min, (5) amnesia less than 24 h, and (6) primary cranial CT imaging. Exclusion criteria were (1) no cranial CT imaging at admission, (2) GCS score < 13, (3) loss of consciousness for 30 min or longer, (4) amnesia for 24 h or longer, and (5) incomplete medical records [3,4].

### 2.2. Patient Screening

Blunt head trauma was defined as (1) any blunt trauma to the head and (2) any craniofacial soft tissue injury, Galea hematoma, craniofacial fracture, or cognitive alteration. The cranial CT was considered positive or negative according to the presence or absence of intracranial hemorrhage (ICH), respectively [3,4]. The incidence of delayed ICH during the hospitalization period or after discharge was evaluated.

Our standard protocol for blunt head trauma included clinical evaluation and an initial cranial CT (CT0). CT of the midface, mandible, and/or the cervical spine was additionally performed in patients with suspicion of trauma-caused fractures of the midface, mandible, and cervical spine after clinical examination. Patients with neurological symptoms, posttraumatic intracranial pathologies, concomitant craniofacial fractures requiring surgical treatment, and patients with unclear home surveillance were admitted for in-hospital observation. Unstable patients were primarily admitted to the intensive care unit (ICU) for stabilization. During hospitalization, the neurological status of the patients was evaluated every hour. A repeat cranial CT imaging (CT1) of patients with pathologic CT0 was performed 8 h after the initial CT scan. A CT1 in patients with normal CT0 was performed in cases of secondary neurological deterioration during in-hospital observation. This was defined as a decrease in GCS score, decrease in level of consciousness, or the development of other focal neurological deficits [3,4]. The length of hospitalization in patients with normal or pathologic CT0 but without concomitant injuries that required surgery was determined for 48 h. In cases of concomitant craniofacial injuries with indication for surgical intervention, the length of stay was prolonged accordingly.

According to the current German guidelines for mTBI, a cranial CT scan is indicated as the “gold standard” in patients with at least one of the following clinical findings: (1) loss of consciousness, (2) definite retrograde amnesia, (3) multiple vomiting, (4) seizures, (5) pathologic neurological signs, (6) clinical signs of cranial fracture, (7) suspicion of impression fracture or penetrating injuries, (8) suspicion of liquorrhea, and (9) coagulopathy or the use of antithrombotic medication (Table 1).

### 2.3. Data Collection

The indication for cranial CT imaging given by the attending resident of oral and maxillofacial surgery after the clinical examination was collected from patients’ hospital charts [3,4]. All CT scans were interpreted by two board certified radiologists. We abstracted all radiological findings that were relevant for an acute intracranial pathology (intracranial hemorrhage, subdural hematoma, epidural hematoma, subarachnoid hemorrhage, impression fractures or penetrating injuries, and hemorrhagic contusion) [3,4]. Intracranial hemorrhage was defined as non-trauma-resulted intraparenchymal hemorrhage, e.g., in cases of pre-traumatic ischemic stroke, in order to differentiate from the trauma-resulted hemorrhagic contusion. The term “impression fracture” describes fractures of the cranial dome with intracranial displacement of the bone fragments after circumscribed direct force. Concomitant post-traumatic injuries of the midface, mandible, and cervical spine were also recorded. Clinical information not reported in the emergency department report was assumed as negative.

Collected data were anonymized before data analysis and comprised patient’s age and gender; antithrombotic medication; trauma etiology; GCS score at initial examination; post-traumatic neurological examination findings indicating a cranial CT (loss of consciousness, amnesia, vomiting, seizures, cephalgia, somnolence, dizziness, and nausea), as well as clinical signs of cranial fracture. The presence of lacerated wounds at the time of admission was also documented; these wounds were defined as injury-related wounds with torn skin, soft tissue, and/or muscle that required primary mono- or multi-layer wound closure at the time of hospital admission.

### 2.4. Statistical Analysis

Data were centralized in electronic format using Microsoft Excel software. Statistical analysis was performed using SAS^®^, Release 9.4 software (SAS Institute Inc., Cary, NC, USA). Patient age was reported as mean and standard deviation (SD), and as a categorical variable (more or less than 65 years). Nominal data were expressed as absolute value (n) and relative prevalence (%). Descriptive statistics were used to describe the baseline patient characteristics. Risk factors were a GCS score < 15, loss of consciousness, amnesia, vomiting, seizures, pathologic neurological signs (cephalgia, somnolence, dizziness, and nausea), clinical signs of cranial fracture, suspicion of impression fracture or penetrating injuries, suspicion of liquorrhea, and the use of antithrombotic medication. Chi-square tests were used to investigate a potential association between risk factors and CT pathologies. The Fischer exact test was used in smaller subgroups. Statistical significance was set at *p* < 0.05. The prevalence of justified and unjustified CT scans was calculated for each investigated variable. Noncompliance was defined as “overperformed” when CT was done, despite the absence of any indicating criteria described in Table 1. The effectiveness of the current German decision rules and GCS score as diagnostic tests were validated by calculating sensitivity, specificity, and positive and negative predictive values using fourfold panels.

## 3. Results

### 3.1. Demographic Distribution

**Figure 1:**
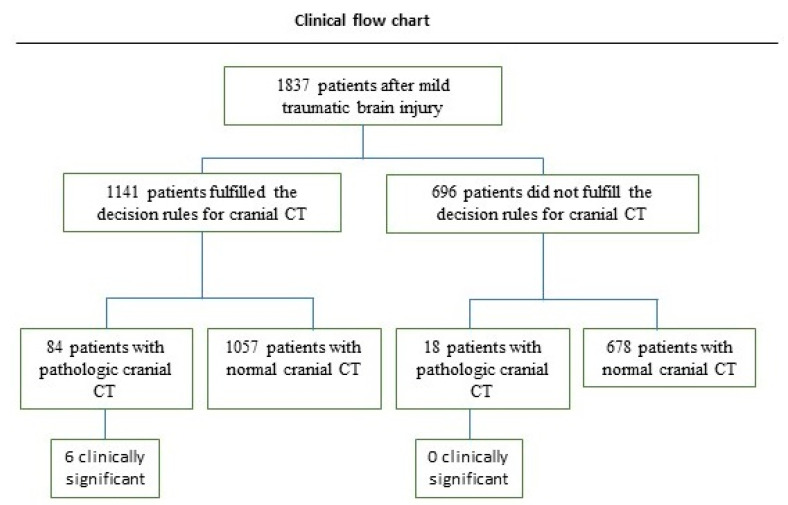
Flowchart showing the assessment of patients who presented to the clinic of oral and maxillofacial surgery after mTBI. CT = computed tomography.

The study collective included 1837 patients with mTBI (Figure 1). There were more males (n = 1.016; 55.3%) than females (n = 821; 44.7%) (male to female ratio = 1.23:1). Patient’s age at the time of trauma ranged from 21 to 106 years, with a mean age of 70.7 years (SD = ±21.10).

Ground-level fall was the most common cause of injury (51.2%). The average GCS score at the initial examination was 15 in 91.1% of the patients, followed by 14 in 5.8% of the patients and 13 in 3.1% of the patients. Neurological symptoms were present 9.9% (n = 183) of patients. Amnesia was the most endorsed symptom (n = 322; 17.5%), followed by loss of consciousness (n = 261; 14.2%) and cephalgia (n = 230; 12.5%). Clinical signs of cranial fracture were present in three (0.1%) patients. Penetrating injuries or cases of liquorrhea were not documented. Lacerated wounds occurred in 61.1% of the patients. Table 2 summarizes the demographic and clinical features of the study cohort.

### 3.2. CT Findings and Patient Outcomes

One hundred and two patients (5.5%) were diagnosed with acute intracranial pathology. A total of 123 intracerebral lesions were radiologically detected, comprising 22 intracranial hemorrhages, 36 subdural hematomas, 9 epidural hematomas, 37 subarachnoid hemorrhages, and 19 contusion hemorrhages. Of these 102 patients, 38.2% (n = 39) were under antithrombotic medication. Skull fracture was detected in 1.0% of the group. Midface and mandible trauma were diagnosed in 30.8% and 4.6% of the patients, respectively. Thirteen cases (0.7%) of cervical spine trauma were also detected. All CT diagnosed post-traumatic injuries are detailed in Table 2.

Ninety-nine out of 102 (97.1%) patients with acute intracerebral lesions were admitted for in-hospital observation (median length of stay: 7 days; SD = ±5). Three of them, one with a subarachnoid hemorrhage, one a subdural hematoma, and one a linear cranial fracture without clinical relevance, refused hospitalization and were discharged home after written consent and clarification about the risks and complications. Most patients with acute intracerebral lesions were male (55.8%; n = 57/102) and older than 65 years (57.8%; n = 59/102). In 41.1% (n = 42/102) of the cases, the injuries were caused by ground-level falls. Eighteen out of the 102 (17.6%) patients presented no neurological symptoms during the initial examination. In total, 61.5% (n = 1130) of the patients were discharged home after initial CT imaging; the majority (n = 1127) had a negative CT. In total, hospital admission for further observation took place for 707 (38.4%) patients (median length of stay: 4 days; SD = ±5). Most inpatients admitted (n = 608; 85.9%) had no radiological alterations at CT0 and 12.9% (n = 79) of them presented neurological symptoms at the initial examination. Forty-one (2.23%) patients were primarily admitted to the intensive care unit due to unstable general conditions.

**Figure 2:**
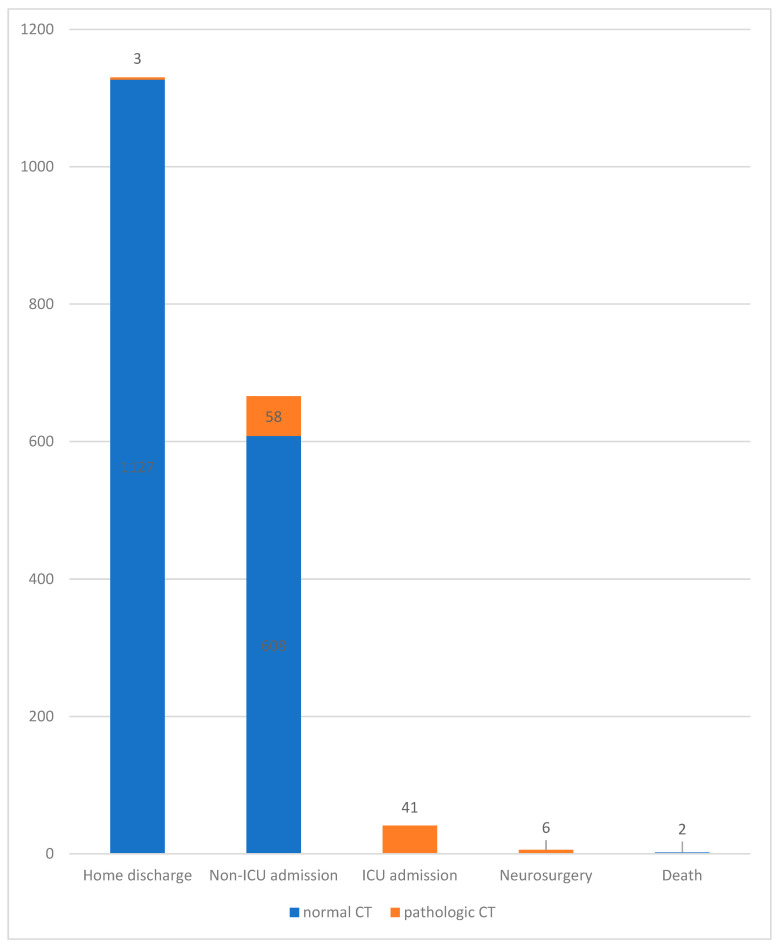
Distribution of patient management und outcomes. ICU = intensive care unit.

Six patients required urgent neurosurgical intervention. We recorded an in-hospital mortality rate of 0.1% (n = 2). The first patient was a 58-year male with presence of cephalgia and dizziness and a normal CT0 but with severe cervical spine trauma. The second one was a 94-year-old female without neurological alterations, a normal CT0, and a concomitant orbital floor fracture. Figure 2 shows the patient’s management and outcome.

### 3.3. Multivariable Analysis

Table 3 shows the distribution of all of the clinical symptoms recorded in correlation with the patient’s age. No significant correlation between these clinical variables and patient age was detected (*p* > 0.05).

**Figure 3:**
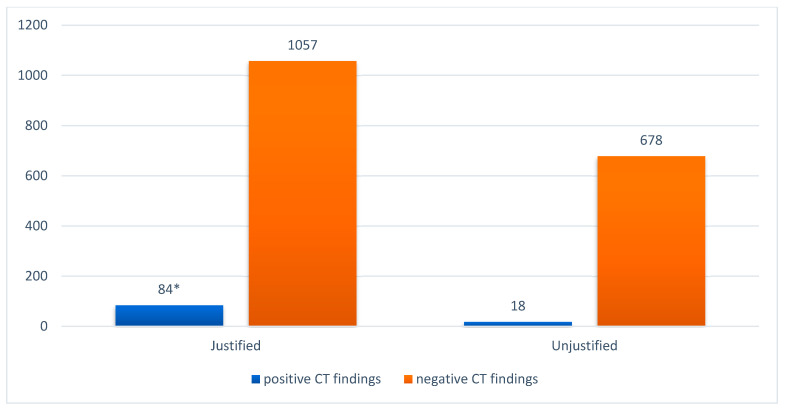
Distribution of justified and unjustified cranial CT scans in association with CT0 findings. CT = computed tomography. * Fischer exact test ≤ 0.0001.

The majority (62.1%) of cranial CT scans included in the sample complied strictly with the guidelines’ decision rules, and 37.8% were not justified and likely avoidable (Table 4). Intracranial lesions were detected in 7.9% of the patients with justified CT scans, and 6 of these patients required neurosurgical intervention. Seventy-three patients received a control CT1 after 8 h. Among the 696 patients with unjustified CT scans, 18 (2.5%) had a pathologic CT0; however, without clinical relevance. A significantly higher incidence of positive cranial CT0 findings was observed in patients with justified CT scans compared with patients with unjustified CT scans (7.9% vs. 2.5%, *p* < 0.0001) (Figure 3). The distribution of all CT pathologies regarding the guideline’s decision rules is presented in Table 4. Intracranial hemorrhage, subdural hematoma, subarachnoid hemorrhage, contusion hemorrhage, and linear skull fracture were significantly more frequently detected in patients with justified CT scans than in patients with unjustified CT scans (*p* < 0.05). No significant difference was observed when diagnosing epidural hematoma, depressed skull fracture, open skull fracture, basal skull fracture, and galea hematoma (*p* > 0.05).

Table 5 shows the distribution of positive cranial CT0 findings regarding the GCS score. Conversely, a decreasing GCS (<15) was significantly related to an increased incidence of all different CT0 pathologies (*p* < 0.05).

Table 6 shows the distribution of cranial CT0 findings concerning the demographic features and clinical symptoms of the study patients. Neither gender nor age were statistically associated with posttraumatic intracranial pathology (*p* > 0.05). Positive CT0 findings were presented significantly more often in patients with loss of consciousness, amnesia, seizures, cephalgia, somnolence, dizziness, nausea, and clinical signs of cranial fractures than in patients without these clinical symptoms (*p* < 0.05). Vomiting and antithrombotic medication were not associated with a higher incidence of positive CT0 findings (*p* > 0.05). No cases of impression fracture, penetrating injuries, or liquorrhea were documented.

The diagnostic performance of the guideline-based decision rules and GCS score in predicting positive CT scans is shown in Table 7. The guideline-based decision rules identified 84 out of 102 cases with pathologic CT, showing an 82.35% sensitivity (95% CI: 73.55–89.19), although with a specificity of 39.08% (95% CI: 36.77–41.42). The GCS score correctly identified only 30 out of 102 cases with pathologic CT with 29.41% (95% CI: 20.80–39.25) sensitivity, but with a high specificity of 92.28% (95% CI: 90.92–93.49). The positive predictive value (7.36 vs. 18.29) and negative predictive value (97.41 vs. 95.70 vs. 97.41) were similar between the decision rules and GCS score, respectively.

## 4. Discussion

The uncritical scanning of patients with mTBI is disadvantageous as it will be time-consuming, costly, and increase radiation exposure [20]. Besides the various clinical decision rules developed internationally to standardize the efficiency of CT indication in these patients, every national health system has its own guidelines and recommendations [2]. Stiell et al. documented differences in health systems worldwide, not only for the CT indication, but also for the physician’s specialty ordering a CT [17]. Previous research was conducted mainly from neurosurgeons, emergency physicians, and radiologists.

This study was conducted in a large trauma center in our region that has adopted the current national head injury guidelines for decision-making on cranial CT indication. We retrospectively reapplied the recommended decision rules to estimate the extent of avoidable cranial CT imaging. In this way, we aimed to estimate the likely avoidable CT imaging. The large sample of patients with this trauma pattern referred primarily to our clinic is explained by the presence of obvious concomitant craniofacial injuries, which had to be surgically addressed by oral and maxillofacial surgeons. Consequently, for cost and time effectiveness, these patients were primarily referred to our emergency department and not to other medical disciplines. To the best of our knowledge, this is the first study to evaluate the compliance of a clinic of oral and maxillofacial surgery with the national guideline’s recommendations for cranial CT indicating in patients following mTBI.

The documented patient’s age and gender makes our study cohort appropriate for comparison with the international literature [8,16,26,27]. Ground-level fall was the most common trauma cause (51.2%), confirming the results of previous studies and highlighting the upcoming social problem of increasing age expectancy worldwide [1,6,9,10]. Regarding the primarily clinical examination, a GCS score of 15 was by far the most frequent, and amnesia was the symptom with the highest prevalence, followed by loss of consciousness and cephalgia. The investigation of the potential correlation between a specific type of concomitant trauma of midface or mandible and intracranial hemorrhage was not considered in this study. However, the documented cervical spine trauma in 13 patients is concerning, and protocols for the simultaneous radiological evaluation of the cervical spine after this trauma pattern should be reconsidered.

We observed pathologic cranial CT findings in 5.5% of patients, likely approximate to the results of previous studies, and subarachnoidal hemorrhage was the most frequent pathology [8,16,26,27]. In addition, the recorded mortality rate of 0.1% within the hospitalization period was significantly lower than in past reports [28].

The study results confirmed our original hypothesis that there was low compliance (62.1%) with the recommended decision rules. This statement was similar to the findings of other authors, who reported compliance rates between 60–80% after evaluating other decision rules [11,29,30]. An additional 37.8% of cranial CT scans, representing more than a third of the collective, was performed despite not being recommended and was identified as “likely avoidable”, similar to previously reported rates of 20–40% [11,29,31]. Thus, we confirm the general opinion that posttraumatic cranial CT imaging after mTBI is substantially overused [19]. The study results showed that clinicians of our department had moderate knowledge regarding the relevant national mTBI guidelines and decision rules as a guide recommendation in daily practice, resulting in increased unjustified CT imaging. Melnick et al. also stated that physicians are often either unaware of the decision criteria or ignore them in clinical practice [29]. Some physicians may justify such a CT overuse for advanced diagnosis of a significant intracranial injury even, though the treatment is nonsurgical [11]. We also agree with Quass et al. that CT overuse is influenced by the physicians and the individual patient factors, as the tendency to overestimate the probability of detecting a significant injury is high [32].

Similar to Arab et al. we support the use of guidelines for mTBI treatment because it rarely misses significant injuries that require neurosurgical intervention [20]. Although several studies have proven the efficacy of the various decision rules in reducing the number of unjustified CT scans, we showed a paradoxical increase in CT performance after implementing our national guideline’s criteria that resulted in increased hospital admissions. This statement argues with that of Stiell et al. [31]. Thus, we think that high compliance does not always equate with more efficient utility.

Our study observed a significantly higher incidence of positive cranial CT0 findings in patients with justified CT scans compared to patients with unjustified CT scans. All 6 study patients who required urgent neurosurgical intervention were correctly indicated for primary CT imaging. Fortunately, none of the patients in the “overperformed” group presented clinically significant findings that otherwise would have been missed. We logically found a significantly higher incidence of intracranial hemorrhage, subdural hematoma, subarachnoid hemorrhage, contusion hemorrhage, and linear skull fracture in patients with justified CT scans. However, no direct comparison can be made to similar studies due to the different protocol and studied variables.

Loss of consciousness, amnesia, seizures, cephalgia, somnolence, dizziness, nausea, and clinical signs of cranial fractures were clinical variables associated with increased cranial CT pathology. These symptoms and clinical findings can be directly and easily derived from an initial structured examination, helping the clinical practice by risk stratification and indicating a primary cranial CT [5,6]. Wu et al. also reported that patients with loss of consciousness were significantly more likely to present intracranial pathologies [33]. Similar to the Canadian CT Head Rule, we also considered amnesia as a symptom with suspicion for brain injury [17]. Our results about focal neurologic deficits, such as cephalgia, somnolence, dizziness, and nausea, as significant risk factors for an abnormal cranial CT are in accordance with previous research [15]. Seizures have not been investigated sufficiently in past studies; however, we considered posttraumatic seizures as a medium risk factor for intracranial abnormalities [15,17]. Regarding vomiting, we did not find a significant correlation with pathologic CT scans. Arab et al. and Borland et al. also did not consider vomiting as a risk factor for a minor head injury, unlike Sadegh et al. and the American College of Emergency Physicians [15,20,34]. Cases of impression fractures, penetrating injuries, or liquorrhea were also not documented; thus, no valid recommendation can be stated. We also found no difference in demographic indicators, such as age and gender. Considering other international decision rules, we support our current national guidelines’ recommendations by not including patient’s age as a high-risk factor for increased intracranial pathology. However, unlike these rules, our results recommend cephalgia as an absolute and not an optional indicator for primary cranial CT after mTBI. The widely-held belief of increased intracranial complications in patients under antithrombotic medication was also not confirmed [5,8,16,26,27]. However, this patient group has to be individually evaluated in further studies considering not only radiographic findings, but also the pharmacological effect of different drugs.

The initial GCS score predicted intracranial CT pathologies in our collective with a high accuracy, which was in accordance with Fournier et al. and Sadegh et al. [15,18]. Consequently, a decreasing GCS score could always justify primary cranial CT imaging as it was significantly related to increased intracranial pathologies. However, the reliability of the GCS score as triage tool has to be further evaluated in future studies considering also other clinical factors and demographic parameters. The incidence of immediate intracranial hemorrhage in patients with normal neurological status is also very concerning, as more than 70% of the study patients with pathologic CT1 appeared with a GCS score of 15 and a normal mental status. GCS score detected five out of six patients who needed a neurosurgical intervention and also intracranial pathology with 29.41% sensitivity and 92.28% specificity. This is in accordance with the findings of Lesko et al. [35]. The high specificity of the GCS score ensures that patients with score of 15 have highly probable a normal cranial CT. However, the low sensitivity has to be considered, as significant pathologic findings could remain undetected, leading to increased CT use.

Furthermore, 44.4% of the patients with pathologic CT0 presented no neurologic alterations at the initial examination, but there was physical evidence of concomitant soft tissue trauma. As most studies did not explicitly investigate the degree of soft tissue injury, we believe that the use of the Injury Severity Score could reliably help with risk stratification. Posttraumatic lacerated wounds were detected in 62.1% of patients; however, the research on their clinical impact was not the aim of this study.

Our results showed a high sensitivity (82.35%) but low specificity (39.08%) of the national guideline-based decision rules for predicting cranial CT pathologies. These criteria had a positive predictive value of 7.36% and a negative predictive value of 97.41%. This model could accurately detect all six patients who needed a neurosurgical procedure. Arab et al. evaluated the Canadian CT Head Rule retrospectively and detected a lower sensitivity but higher specificity related to these criteria [20]. These variations could be related to the smaller collective of the study of Arab et al., the different study protocol, and the different education level of physicians [20].

Our study did not compare the compatibility of the applied rules with other international decision tools. Although the predictor variables of this study were well standardized, no assessment of their interobserver agreement was made, and other potentially valuable features, such as alcohol or drug intoxication and high-energy trauma, were not assessed. We think that examination by suspected intoxication could be unreliable for appropriate risk stratification in daily practice by presenting suspected neurological deficits, which otherwise would not exist without prior consumption. Therefore, we suggest optional CT imaging by intoxicated patients with a decreased GCS score after careful clinical assessment, thus remaining in line with the recommendations of the national guidelines. Previous studies considered clinical signs of basal skull fracture, such as rhinorrhoea, otorrhea, Battle’s sign, raccoon’s eye, and hemotympanum, are of great relevance for minor head injury. Our study did not examine these clinical symptoms separately and could be aim of future research [15,17].

We agree with Tan et al. that the lack of consistently highly sensitive decision rules in the international literature may have caused physicians to have a low tolerance for missed potential life-threatening intracranial pathologies [11]. This fact has made them hesitant to rely solely on the decision rules instead of using their judgment while assessing patients [11]. We believe that the format of the decision rules followed in our clinic, a simple list of variables, makes its application clinically sensible for the intended audience of busy emergency physicians, at least in our trauma center. The high sensitivity for significant outcomes requiring intervention or in-hospital observation guarantees a pragmatic and safe approach to patient management. It is also acceptable and specific, making it an efficient tool for clinicians. However, the true value of these rules should be verified in prospective trials that assess the accuracy, interobserver agreement, clinician acceptability, and potential impact on a new, larger, and multivariable patient population.

In summary, we anticipate that cranial CT would be judged mandatorily for patients with any of GCS score < 15, loss of consciousness, amnesia, seizures, cephalgia, somnolence, dizziness, nausea, or clinical signs of cranial fractures. These clinical features should assist clinicians of different disciplines in daily practice in decision making, especially in emergency departments without possibilities for 3D imaging. We underline the importance of continuing medical education as well as adequate communication between physicians, patients, and their relatives. Considering our results, we think there is still ample room for improvement in correctly applying the recommended national decision rules in our clinic. Thus, we believe that a significant reduction in cranial CT use could be safely achieved. Nonetheless, we intend to validate the investigated rules and our findings in future research.

An important strength of the current study is that all patients with mTBI were selected without drop-out, and represented a wide spectrum of demographic and clinical characteristics increasing generalizability regarding CT overuse. Nevertheless, we acknowledge some limitations. First, the retrospective nature of data collection could lead to documentation bias, especially on clinical symptoms. Second, a control group of patients without cranial CT imaging after mTBI was absent and the missing of direct comparison could limit the external validity of our study. A retrospective data extraction of patients with mTBI who were referred to our hospital but not received a cranial CT was not feasible. We assumed that applying the guideline’s criteria in the non-CT group would have led to the unintended consequence of increasing CT imaging. Third, this study was conducted only in patients referred to the emergency department of an oral and maxillofacial surgery clinic of a single trauma center. Other departments of different specialties or hospitals could have managed these patients differently or following other decision rules according to the institution policy, country, and health system. This important limitation could have significant impact in the external validity of our study conclusions. Fourth, the time period between the trauma event and the in-hospital clinical evaluation and the initial CT scan was not documented in emergency reports, which could have affected the primary outcome. Future prospective research with an international multicenter design should help to develop standardized clinical decision support for reducing CT scanning in likely avoidable circumstances.

## 5. Conclusions

We demonstrate the feasibility of the current German guideline-based decision rules after mild traumatic brain injury to diagnose intracerebral pathologies and appropriately reduce unnecessary cranial CT imaging. The compliance of our clinic with the guidelines recommendations was low, and more than 1/3 of the performed CT scans were identified as “likely avoidable”. The incidence of intracranial pathology in patients with justified CT scans was significantly higher compared with patients with unjustified CT scans. Of the decision rules, GCS score < 15, loss of consciousness, amnesia, seizures, cephalgia, somnolence, dizziness, nausea, and clinical signs of cranial fractures were associated with intracranial pathology. The investigated decision rules showed a high sensitivity but low specificity for predicting CT pathologies; however, all study patients with clinical significance who required urgent neurosurgical intervention were detected. Continuing medical education would reduce noncompliance with the decision rules. Future multicenter studies should develop standardized clinical decision rules in order to define best practices in risk/benefit outweighing.

## Figures and Tables

**Table 1:** Clinical criteria indicating a cranial CT according to the German guidelines after mild traumatic brain injury.

Criteria
Loss of consciousness
Amnesia
Multiple vomiting
Seizures
Pathologic neurological signs (cephalgia, somnolence, dizziness, and nausea)
Clinical signs of cranial fracture
Impression fracture or penetrating injuries of the skull
Suspicion of liquorrhea
Coagulopathy or use of antithrombotic medication

**Table 2:** Baseline characteristics of the overall study population after mTBI.

	Study Population	
	n	%
Total	1837	100.00%
**Demographics**		
Age ≥ 65 years	1062	57.81%
Age < 65 years	775	42.19%
Male sex	1016	55.31%
Female sex	821	44.69%
**Trauma etiology**		
Ground-level fall	941	51.22%
Fall from height	21	1.14%
Stair fall	19	1.03%
Traffic accident	30	1.63%
Bicycle	116	6.31%
Epileptic fall	17	0.93%
Fall after syncope	154	8.38%
Violence	292	15.90%
Sport	20	1.09%
Fall after alcohol consume	185	10.07%
Other mechanism	42	2.29%
GCS scale		
15	1673	91.07%
14	106	5.77%
13	58	3.16%
**Clinical examination**		
Loss of consiousness	261	14.21%
Cephalgia	230	12.52%
Amnesia	322	17.53%
Vomiting	45	2.45%
Neurological signs	183	9.96%
Seizures	17	0.93%
Clinical signs of cranial fracture	3	0.16%
Impression fracture or penetrating injuries	0	0.00%
Suspicion of liquorrhea	0	0.00%
Lacerated wounds	1141	62.11%
**CT findings**		
Intracranial hemorrhage	22	1.20%
Subdural hematoma	36	1.96%
Epidural hematoma	9	0.49%
Subarachnoidal hemorrhage	37	2.01%
Contusion hemorrhage	19	1.03%
Depressed skull fracture	3	0.16%
Linear skull fracture	9	0.49%
Open skull fracture	2	0.11%
Basal skull fracture	6	0.33%
Galea hematoma	203	11.05%
Midface trauma	567	30.87%
Mandible trauma	85	4.63%
Cervical spine trauma	13	0.71%
Dental trauma	45	2.45%

**Table 3:** Distribution and statistical analysis of the clinical symptoms in association with patient’s age.

Criteria	Study Population	Age < 65 Years (n/%)	Age ≥ 65 Years (n/%)	*p*-Value
Loss of consciousness	261	112 (14.45%)	149 (14.03%)	* 0.7983
Cephalgia	230	99 (12.7%)	131 (12.3%)	* 0.7758
Amnesia	322	141 (18.19%)	181 (17.04%)	* 0.5219
Multiple vomiting	45	18 (2.32%)	27 (2.54%)	* 0.7634
Seizures	17	9 0.75%)	8 (1.16%)	* 0.3671
Pathologic neurological signs (somnolence, dizziness, nausea)	183	78 (10.06%)	105 (9.89%)	* 0.9002
Clinical signs of cranial fracture	3	2 (0.26%)	1 (0.09%)	** 0.5767
Impression fracture or penetrating injuries	0	0	0	***
Suspicion of liquorrhea	0	0	0	***
Coagulopathy or use of antithrombotic medication	696	281 (36.26%)	415 (39.08%)	* 0.2187

Abbreviations: n = number; % = percentage. * Chi-square Fischer’s exact test. ** Fischer’s exact test. *** not applicable.

**Table 4:** Justification of primary cranial CT imaging according to the guideline-based clinical criteria in relation to the CT0 findings.

CT Findings		Total	Justified 1141 (62.11%)		Unjustified 696 (37.88%)		*p*-Value
			n	%	n	%	
Intracranial hemorrhage	positive	22	19	1.03%	3	0.16%	* 0.0183
	negative	1815	1122	61.08%	693	37.72%	
Subdural hematoma	positive	36	30	1.63%	6	0.33%	* 0.0080
	negative	1801	1111	60.48%	690	37.56%	
Epidural hematoma	positive	9	8	0.44%	1	0.05%	** 0.1659
	negative	1828	1133	61.68%	695	37.83%	
Subarachnoidal hemorrhage	positive	37	29	1.58%	8	0.44%	* 0.0394
	negative	1800	1112	60.53%	688	37.45%	
Contusion hemorrhage	positive	19	18	0.98%	1	0.05%	* 0.0032
	negative	1818	1123	61.13%	695	37.83%	
Depressed skull fracture	positive	3	3	0.165	0	0%	** 0.2936
	negative	1834	1138	61.95%	696	37.89%	
Linear skull fracture	positive	9	9	0.49%	0	0%	** 0.0161
	negative	1828	1132	61.62%	696	37.89%	
Open skull fracture	positive	2	2	0.11%	0	0%	** 0.5291
	negative	1835	1139	62.0%	696	37.89%	
Basal skull fracture	positive	6	6	0.33%	0	(0%)	** 0.0890
	negative	1831	1135	61.79%	696	37.89%	
Galea hematoma	positive	203	125	6.80%	78	4.25%	* 0.8675
	negative	1634	1016	55.31%	618	33.64	

n = number; % = percentage. * Chi-square Fischer’s exact test. ** Fischer’s exact test.

**Table 5:** Distribution and statistical analysis of the CT findings in relation to the GCS score.

CT Findings	Total	GCS = 13/14 164 (8.93%)		GCS = 15 1673 (91.07%)		*p*-Value
		n	%	n	%	
Intracranial hemorrhage	22	10	6.09%	12	0.71%	* <0.0001
Subdural hematoma	36	12	7.31%	24	1.43%	* <0.0001
Epidural hematoma	9	4	2.43%	5	0.29%	** 0.0054
Subarachnoidal hemorrhage	37	9	5.48%	28	1.67%	* 0.0009
Contusion hemorrhage	19	6	3.65%	13	0.77%	** 0.0047
Depressed skull fracture	3	2	1.21%	1	0.05%	** 0.0224
Linear skull fracture	9	4	2.43%	5	0.29%	** 0.0054
Open skull fracture	2	2	1.21%	0	0%	** 0.0079
Basal skull fracture	6	5	3.04%	1	0.05%	** 0.0000
Galea hematoma	203	10	6.09%	193	11.53%	* 0.0340

n = number; % = percentage; GCS = Glasgow Coma Scale. * Chi-square Fischer’s exact test. ** Fischer’s exact test.

**Table 6:** Cranial CT0 findings in relation to the demographics and clinical features after mTBI.

Characteristics		Positive CT Findings 102 (5.55%)		Negative CT Findings1735 (94.45%)		*p*-Value
		n	%	n	%	
Gender	Male	57	5.61%	959	94.39%	* 0.9044
	Female	45	5.48%	776	94.51%	
Age	≥65 years	59	5.56%	1003	94.44%	* 0.9947
	<65 years	43	5.55%	732	94.45%	
GCS score	13/14	30	18.29%	134	81.71%	* <0.0001
	15	72	4.30%	1601	95.70%	
Loss of consciousness	Yes	39	14.94%	222	85.06%	* <0.0001
	No	63	4.0%	1513	96.0%	
Amnesia	Yes	31	9.63%	291	90.37%	* 0.0004
	No	71	4.69%	1444	95.31%	
Multiple vomiting	Yes	3	6.67%	42	93.33%	** 0.7360
	No	99	5.52%	1693	94.48%	
Seizures	Yes	4	23.52%	13	76.48%	* 0.0122
	No	98	5.38%	1722	94.62%	
Cephalgia	Yes	32	13.91%	198	86.09%	* <0.0001
	No	70	4.36%	1537	95.64%	
Somnolence	Yes	9	16.36%	46	83.64%	** 0.0026
	No	93	5.22%	1689	94.78%	
Dizziness	Yes	29	16.48%	147	83.52%	* <0.0001
	No	73	4.39%	1588	95.61%	
Nausea	Yes	9	12.16%	65	87.84%	** 0.0189
	No	93	5.28%	1670	94.71%	
Clinical signs of cranial fracture	Yes	3	100%	0	0%	** 0.0002
	No	99	5.39%	1735	94.61%	
Impression fracture or penetrating injuries	Yes	0	0	0	0	***
	No	0	0	0	0	
Suspicion of liquorrhea	Yes	0	0	0	0	***
	No	0	0	0	0	
Coagulopathy or use of antithrombotic medication	Yes	39	5.97%	614	94.03%	* 0.5594
	No	63	5.32%	1121	94.68%	

n = number; % = percentage. * Chi-square Fischer’s exact test. ** Fischer’s exact test. *** not applicable.

**Table 7:** Accuracy of guideline-based decision rules and GCS score in predicting cranial CT findings.

	Sensitivity (95% CI)	Specificity (95% CI)	PPV (95% CI)	NPV (95% CI)
**Guidelines-based** **Decision rules**	82.35 (73.55–89.19)	39.08 (36.77–41.42)	7.36 (5.91–9.03)	97.41 (95.94–98.46)
GCS	29.41 (20.80–39.25)	92.28 (90.92–93.49)	18.29 (12.70–25.07)	95.70 (94.61–96.62)

GCS = Glasgow coma scale; CT = computed tomography; CI = confidence interval; PPV = positive predictive value; NPV = negative predictive value.

## Data Availability

The data presented in this study are available on a reasonable request from the corresponding author.
